# Exploring reasons for usage discontinuation in an internet-delivered stress recovery intervention: A qualitative study

**DOI:** 10.1016/j.invent.2023.100686

**Published:** 2023-10-24

**Authors:** Auguste Nomeikaite, Odeta Gelezelyte, Thomas Berger, Gerhard Andersson, Evaldas Kazlauskas

**Affiliations:** a Center for Psychotraumatology, Institute of Psychology, Vilnius University, Vilnius, Lithuania; b Department of Clinical Psychology and Psychotherapy, University of Bern, Switzerland; c Department of Behavioural Sciences and Learning, Department of Biomedical and Clinical Sciences, Linköping University, Linköping, Sweden; d Department of Clinical Neuroscience, Karolinska Institute, Stockholm, Sweden

**Keywords:** Internet intervention, Usage attrition, Stress recovery, Healthcare workers

## Abstract

Internet-delivered cognitive behavioral therapy (ICBT) interventions can be as effective as traditional face-to-face therapy for various mental health conditions. However, a significant challenge these online interventions face is the high rate of people who start but then stop using the program. This early discontinuation can be seen as incomplete treatment and can reduce the potential benefits for users. By exploring why people stop using ICBT programs, we can better understand how to address this problem. This study aimed to examine the experiences of healthcare workers who had stopped using a therapist-guided internet-delivered stress recovery program to gain deeper insights into usage attrition. We conducted semi-structured interviews with twelve participants who were female healthcare workers ranging in age from 24 to 68 years (*M* = 44.67, *SD* = 11.80). Telephone interviews were conducted and the data were transcribed and analyzed using thematic analysis. Qualitative data analysis revealed that most participants had multiple reasons for discontinuing the program. They identified both barriers and facilitators to using the program, which could be categorized as either personal or program related. Personal aspects included life circumstances, personal characteristics, and psychological responses to the program. Program-related aspects encompassed technical factors, program content, and the level of support provided. The findings of this study can enhance our understanding of why people stop using guided internet-delivered programs. We discuss the practical and research implications, with the ultimate aim of improving the design and efficacy of internet interventions.

## Highlights

• Most healthcare workers had multiple reasons for discontinuing the internet-delivered stress recovery program.
• Personal and program-related barriers and facilitators of program usage have been identified.
• Personal usage aspects included life circumstances, personal characteristics, and psychological responses to the program.
• Program-related usage aspects included technical factors, program content, and the level of support provided.

## 1 Introduction

The internet has changed the way we approach mental health care by enabling innovative internet-delivered treatments that can be just as effective as traditional face-to-face therapy (Hedman-Lagerlöf et al., 2023). To date, most of the research has focused on internet-delivered cognitive behavioral therapy (ICBT), which was found to be effective in improving different mental health aspects (Hedman-Lagerlöf et al., 2023), such as depression (Andersson and Berger, 2021), stress (Heber et al., 2017), anxiety (Andersson et al., 2019). However, such internet interventions, and in particular when there is limited therapist contact, are often characterized by high usage attrition rates, which constitute the phenomenon where a participant stops using the program (Eysenbach, 2005). Research shows that almost half of those who start the treatment do not take part in the full ICBT program (Cross et al., 2022; Waller and Gilbody, 2009). Overcoming this issue is crucial, as early discontinuation of psychological treatment can reduce its potential outcomes (Robinson et al., 2020).

People may discontinue using internet mental health interventions for various reasons. Firstly, the use of such interventions may vary depending on the individual's motivation (Alfonsson et al., 2016; Lawler et al., 2021), specific needs, and preferences (Johansson et al., 2015). While some people may find them helpful, others may prefer face-to-face interactions or other traditional therapy methods (Holst et al., 2017; Lawler et al., 2021), and a mismatch between these needs can lead to an early dropout (Gonzalez Salas Duhne et al., 2022). In addition, unmet expectations (Johansson et al., 2015) and different life circumstances that the participants face, e.g., lack of time (Waller and Gilbody, 2009), may also affect use of such programs. Moreover, adherence may be reduced by the fatigue that the user experiences due to the treatment burden (Heckman et al., 2015). It is worth noting that participants may discontinue using the program also due to changes in their well-being, including both deterioration (Fenski et al., 2021; Johansson et al., 2015) or a rapid improvement (Arndt et al., 2020). Thus, the reasons for discontinuing the use of internet interventions can often be related to contextual or personal characteristics.

ICBT program-related specific intervention factors may also contribute to usage attrition. First, there is evidence that guided online interventions have higher rates of program module completion compared to unguided interventions (Baumeister et al., 2014). The absence of a human connection and difficulties in establishing therapeutic alliance in internet interventions can be significant factors for usage discontinuation (Berger, 2017). A recent meta-analysis on RCTs showed that human contact before and during internet intervention could significantly increase the effects of treatment for depression and decrease dropout (Krieger et al., 2023). In addition to therapist guidance, persuasive technology elements, such as email reminders, can also be effective (Kelders et al., 2012). Nevertheless, the lack of personalization and tailored support in internet interventions can be a drawback for some individuals who require more individualized care (Johansson et al., 2015; Rozental et al., 2015). Moreover, technical difficulties, such as poor computer literacy or issues with the platform itself, can hinder the user experience and discourage people from continuing engagement in internet interventions (Lawler et al., 2021). Finally, it is known that internet interventions can lead to negative effects for a small proportion of its users (Rozental et al., 2015), and it is possible that such effects can at least be partly related to the program format and content (Johansson et al., 2015; Lawler et al., 2021). Overall, it is important to find out what factors are important in increasing participation and reducing dropout in internet interventions of various intensities and targeting different areas of mental health.

Healthcare workers face difficult working conditions that can lead to mental health problems such as burnout, anxiety, and depression (Mira et al., 2020; Sani et al., 2022). However, long working hours, hectic schedules, and the prevailing stigma of reaching out for psychological help can make regular face-to-face therapy difficult (Knaak et al., 2017). Internet interventions could help overcome these barriers to seeking psychological help and have been proven to be effective in improving some components of well-being in medical professionals (Smoktunowicz et al., 2021). The “For Recovery from Stress” (FOREST) internet intervention is a 6-week ICBT program with a focus on mindfulness, tailored for healthcare workers, and developed in close collaboration with experts from the healthcare system (Jovarauskaite et al., 2021). A previous randomized controlled trial (RCT) showed that the FOREST program was effective in helping nurses in developing stress recovery skills, reducing levels of perceived stress, depression, and anxiety, as well as increasing overall psychological well-being (Dumarkaite et al., 2023). Further research showed that the updated program FOREST+ was also effective for a broader sample of healthcare workers (Nomeikaite et al., 2023). In these two controlled trials, only about half of the FOREST/FOREST+ users completed the full 6-week program. More research is therefore needed to investigate the specific barriers faced by participants who have stopped using the program.

To improve the development and updating of ICBT programs, it is essential to actively involve clients by seeking and incorporating their feedback. By examining why participants drop out, researchers and clinicians may gain valuable insights that can be useful for intervention design, engagement strategies, and retention. The aim of this qualitative study was to analyze the experiences of healthcare workers who had discontinued an internet-delivered stress recovery program. For this purpose, a semi-structured interview was developed based on previous qualitative research on the experiences of ICBT participants (Biliunaite et al., 2021; Johansson et al., 2015; Lawler et al., 2021). Two main objectives were established: first, to examine the underlying reasons behind the usage discontinuation of an internet-delivered stress recovery program specifically designed for healthcare workers, and second to examine the barriers and facilitators encountered by users of the program.

## 2 Methods

### 2.1 Study design

The qualitative study is associated with a two-armed randomized controlled trial assessing the effectiveness of a tailored internet-delivered stress recovery intervention. The trial was registered on www.clinicaltrials.gov (NCT05553210) and was approved by Vilnius University Psychology Research Ethics Committee (Reference No. 2021-03-22/61). All participants provided informed consent to take part in both the quantitative and qualitative research at the pre-intervention assessment. The current qualitative study was reported following the COREQ (Consolidated Criteria for Reporting Qualitative Research) guidelines.

### 2.2 Participants

Healthcare workers were invited to participate in the internet intervention through national media, social network groups, healthcare institutions, and email databases. The RCT involved 91 healthcare workers who were randomly assigned to two study groups (ratio 1:1): 1) standard intervention plan group (SIP; *n* = 45), 2) tailored intervention plan group (TIP; *n* = 46). Participants who started but then stopped using the program were included in the qualitative study. Eligibility criteria: 1) completing fewer than 4 modules (< 4 weeks), 2) at least one login to the program. Researcher AN manually reviewed the login details of all participants to the program modules and screened eligible participants for the study. A total of 24 participants had dropped out of the program by week four, 4 of whom have never logged in to the program. Of the 20 participants that met eligibility criteria, 12 were interviewed (*n*_SIP_ = 6; *n*_TIP_ = 6). Participants who did not agree to participate in qualitative interviews indicated that they did not have time for it or had little involvement in the program and therefore did not want to talk about it. The study flow is presented in Appendix 1.

The characteristics of the participants included in the qualitative study are presented in Table 1. The sample comprised 12 women aged 24–68 (*M* = 44.67, *SD* = 11.80). More than half of those included (*n* = 7) in the study were nurses or assistant nurses (58 %), three of them were doctors of medicine (25 %), and two were clinical psychologists (17 %). No significant differences were found between subjects included and subjects excluded from the qualitative study (Appendix 2).

**Table 1:** Participant characteristics (*N* = 12).

ID	Age	Gender	Profession (field)	Modules opened	Login count	Feedback received	Group assignment	Intervention plan
P1	20	Female	Dental assistant (outpatient)	3	2	3	TIP	Twice a week
P2	43	Female	General practitioner, M.D. (outpatient)	1	2	2	SIP	–
P3	52	Female	Dental technician, M.D.	1	1	2	TIP	Once a week
P4	43	Female	General practice nurse (outpatient)	1	5	2	TIP	Each workday
P5	44	Female	General practice nurse (nursing)	2	2	2	TIP	Twice a week
P6	52	Female	General practice nurse (inpatient)	3	3	4	SIP	–
P7	64	Female	Clinical psychologist (outpatient)	1	1	0	TIP	Twice a week
P8	35	Female	Clinical psychologist (inpatient, rehabilitation, nursing)	1	2	1	SIP	–
P9	29	Female	Dietitian, M.D. (outpatient)	3	5	3	SIP	–
P10	45	Female	General practice nurse (rehabilitation)	3	5	4	TIP	No SMS reminders
P11	24	Female	Assistant nurse (inpatient)	2	3	2	SIP	–
P12	46	Female	General practice nurse (outpatient)	2	5	2	SIP	–

Note. SIP – standard intervention plan group, TIP - tailored intervention plan group.

### 2.3 Intervention

The 6-week therapist-supported internet intervention CBT program FOREST+ for healthcare workers (Nomeikaite et al., 2023), which is a modification of FOREST (Jovarauskaite et al., 2021) intervention for nurses, comprised six modules, delivered on a weekly schedule (see Table 2). Each program module included psychoeducational texts, relaxation instructions, worksheets, video, and audio recordings. At the end of each module, participants received short feedback from their therapist. Participants had the possibility to contact their therapist by messages within the program. In addition, users of the tailored intervention plan group set a plan of how much they intended to use the program and how many short message reminders they would need (from none to two per day). The intervention plan was drawn up during a brief telephone interview with a researcher at pre-intervention. The standard intervention plan group used the program without a tailored intervention plan and did not receive any additional message reminders. Both groups received email reminders when the new module was opened and once again to complete it if they had not done so. Participants were also contacted by the study administrators for a short telephone interview during and after the program to encourage them to use the program and to answer any technical questions they may have.

**Table 2:** Description of the program modules.

Week	Module	Description
1	Introduction	Introduction to the program and how to proceed; psychoeducation on stress and recovery from stress; assessment of stressors faced and burnout symptoms; breathing relaxation.
2	Psychological detachment	Psychoeducation on body relaxation and improving sleep quality; body tension assessment; body scanning and sleep relaxations.
3	Distancing	Psychoeducation on intrusive thoughts and distancing from work during leisure time; mindfulness and walking meditations; worksheets for intrusive thoughts and distancing.
4	Mastery	Psychoeducation on stress-reducing activities and mastery; worksheet to set active and less active leisure activities; relaxation and a brief body stretching exercise.
5	Control	Psychoeducation on the importance of work/rest balance and self-care; worksheets for identifying current needs and obstacles of recreation.
6	Keeping the change alive	A brief summary of the program and psychoeducation on how to sustain changes in well-being; worksheets to identify what is most helpful for stress recovery; brief relaxation.

Participants' usage of the program is shown in Table 1. All participants have opened at least one program module: 5 (41.7 %) opened one module, 3 (25.0 %) – two modules, and 4 (33.3 %) – three modules. The number of user logins to the program varied from 1 to 5. Participants received 0 to 4 feedback messages from their therapists during the program. However, none of them tried to contact the therapists.

### 2.4 Semi-structured interview

The semi-structured interview protocol was developed by the authors of this study (see Appendix 3). The interview structure was based on the analysis of literature, which revealed several broad topics: participant expectations for the program, motivation to engage, experience of using the program (format, content, support, and reminders), life circumstances, and personal characteristics that may have influenced the use of the program. The semi-structured interview comprised 10 mandatory questions and 20 prompting questions divided into four categories: (A) overall experience of using the program (3 mandatory questions (*n*_*m*_ = 3), 6 prompting questions *n*_*p*_ = 6), e.g., *What do you think made you stop using the program?*); (B) factors related to the program and to its use (*n*_*m*_ = 3, *n*_*p*_ = 9; e.g., *How do you feel about the fact that the program was implemented online?*); (C) personal characteristics and life circumstances (*n*_*m*_ = 1, *n*_*p*_ = 3; e.g., *To what extent might any circumstances in your life have influenced your use of the program?*); (D) recommendations and other observations (*n*_*m*_ = 3; e.g., *How do you think we could improve the FOREST+ program?*). The protocol provided the interviewer with a flexible interview structure with guidelines for prompting questions or phrases (e.g., *Tell me more about it*). For each question, an area was marked to highlight information that had already been heard so that the interviewer would not repeat questions. In addition, the interviewer had space in the interview sheet to take notes during the interview. Interviewers also had the opportunity to ask open-ended prompting questions of their own devising if they felt the interviewee had not adequately covered the topic and there was no such question in the protocol.

### 2.5 Procedure

Semi-structured interviews were conducted by telephone from 5 to 16 December 2023 and lasted between 10 and 34 min (*M* = 20.08, *SD* = 7.30). Prior to this, a call was made to arrange a suitable time for the participants to be interviewed so that they were in a private place where they could safely share their experiences. During the main interview, the interviewer introduced themselves as the program researcher and his/her name. The participants were informed that their honest answers were very important for the development and research of the stress recovery program for HCWs. Interviews were audio recorded. Consent to record the interview was obtained before the interview was started. The interviews ended when the interviewer felt that the topic of dropout from the program had been sufficiently covered and when the information obtained was repeated, or when the interviewee expressed that they had nothing more to add.

### 2.6 Characteristics of interviewers, researchers, and auditors

The interviews were conducted by trained researchers (junior researcher and clinical psychologist AN and 4 supervised and trained master students of clinical psychology). Given that AN contributed to the development of the program and studied the outcomes of the FOREST+ program as part of her dissertation research, AN only conducted the first pilot interview with one of the participants and did not conduct further interviews herself. Also, the master's program student interviewers were involved in the research process of the program as a part of their master's thesis but had no direct contact with the participants before the interviews. AN (MSc, with a background in internet interventions and mental health of healthcare workers research; female) and DZ (MSC, clinical psychologist, non-program related researcher; male) conducted the thematic analysis. For both coders, this was the first qualitative study conducted. However, the team of auditors was made of two senior researchers that are leading researchers in the field of internet interventions (TB, PhD, and GA, PhD), and of two senior researchers, who are experienced in qualitative research (EK, PhD, and OG, PhD). Thus, the research group had strong base for discussing the content of the interviews.

### 2.7 Data analysis

The data analysis was based on the thematic analysis method as described by Braun and Clarke (2006), which suggests that more general themes should be extracted from smaller pieces of information. Based on Knox and Burkard's (2009) qualitative interviewing principles, an attempt was made to find consensus among the researchers on the codes, themes, and sub-themes identified. AN and DZ coded the transcribed interviews. AN coded the interviews first by identifying the codes, and then DZ coded all the interviews based on the coding system proposed by AN, with the possibility of modifying or supplementing these codes, by common decision. All data coding was done using the ATLAS.ti. Each coder grouped the final codes that derived from the interviews into meaningful themes and sub-themes, as well as selected the quotes that best reflected them. This was followed by a discussion of the themes and sub-themes. To help reach consensus, the discussion was moderated by OG. This process was later reviewed by senior researchers EK and TB, and the model was adjusted in the light of feedback until consensus with all team members was reached.

## 3 Results

### 3.1 Reasons for discontinuing participation

**Fig. 1:**
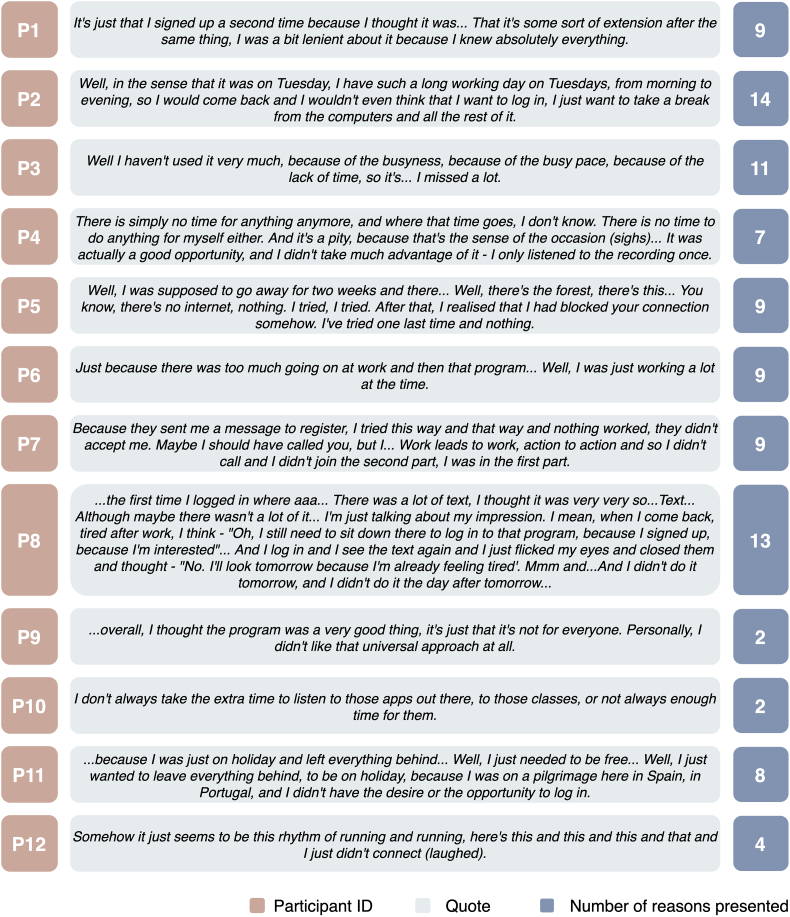
Reasons for stopping participation in an internet-delivered intervention for stress recovery.

The results of the qualitative analysis of the first mentioned reasons and the number of times the reasons for discontinuing participation in the internet-delivered stress recovery intervention were mentioned are presented in Fig. 1. The number of reasons given by participants varied from 2 to 14 (*M* = 8.05, *SD* = 3.68). The most common first-mentioned reason given by five participants (P3, P4, P6, P10, P12) was lack of time and busy pace in life. Three participants indicated that they did not like the format or nature of the program (P2, P8, P9). Few participants encountered technical difficulties when accessing the program (P5, P7). Several participants were also unable to use the program because they were away at the time (P5, P11). Two participants indicated that they felt too fatigued to use the program after work (P2, P8). Finally, one participant stated that she had stopped using the program because she had already participated in the program before (P1).

### 3.2 Barriers and facilitators

**Fig. 2:**
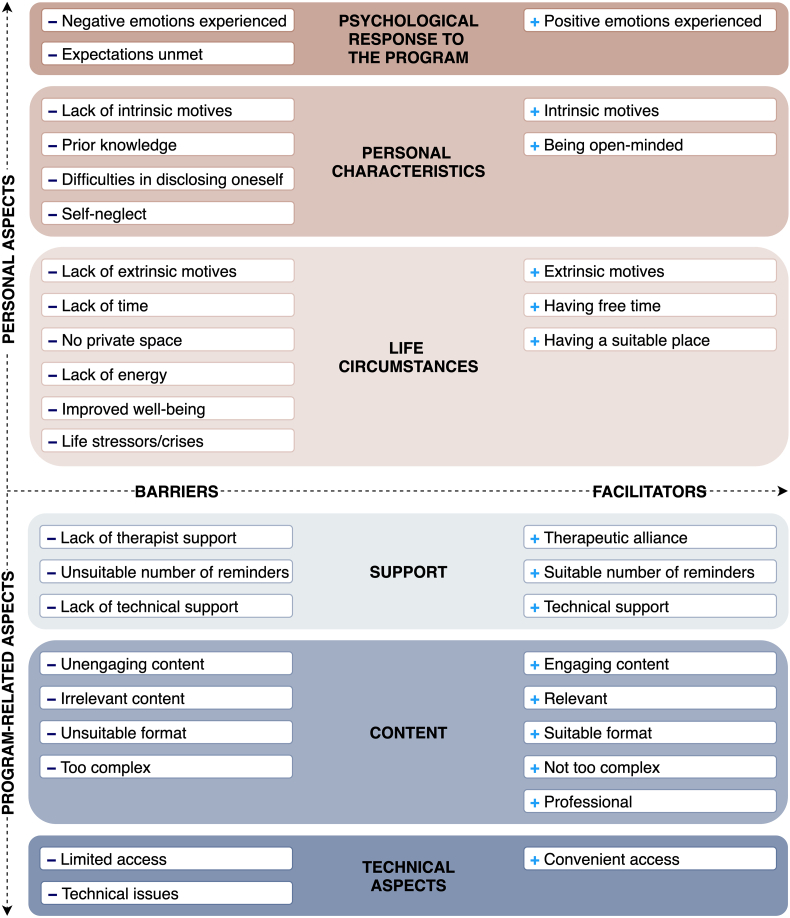
Barriers and facilitators of participation in an internet-delivered intervention for stress recovery.

The full model of barriers and facilitators in relation to program use is presented in Fig. 2. In total, six key themes were identified. Three of these themes were attributed to the personal aspects dimension and the remaining three – to the program-related dimension. For each theme, facilitators and barriers to the use of the program were distinguished. In Appendix 4 we report quotes for each sub-theme and codes.

#### 3.2.1 Psychological response to the program

A theme named psychological response to the program emerged during qualitative analysis, reflecting participants' subjective experience of the program. Some participants experienced negative emotions while using the program, such as anxiety, irritability, anger, guilt, loneliness, sadness, and self-pity, which acted as a barrier to continue participation. For example, one participant who indicated that she was currently being treated for depression stated that during the program she experienced: “*Such disappointment with my own... It is a bit disappointing that I can't manage my time so that I can't find fifteen minutes or half an hour for it.*” [P4]. Meanwhile, other participants noted positive experiences using the program, such as optimism and better self-awareness.

Participants were also asked about their expectations regarding the program and if they were met. We noted that some participants stopped using the program because it did not meet their expectations of having a live interaction with a therapist: “*Maybe my expectations were higher and just too high. I was expecting to see someone who would explain, who would... It would be interesting to listen to, and it's so... It's dull like a textbook.”* [P6]. Other expectations indicated by the participants were that the program could be more interesting and engaging, more professional, and more able to help them improve their well-being.

#### 3.2.2 Personal characteristics

Certain personal characteristics were identified as barriers or facilitators. Users indicated that a lack of motivation led to non-use of the program, while others felt motivated to use the program for reasons such as the desire to help themselves or even professional interest: “*I think it started during the pandemic, and I think it's the third time that I've shared it and then I thought I should register myself and see what you're offering and what it's all about and I'd be interested in that too... In terms of information and maybe something for myself or my work.”* [P8].

Existing knowledge about stress reduction methods also hindered the use of the program, as participants felt that the information presented was already known to them. In addition, the use of the program was complicated by personal characteristics such as a lack of concern for one's own well-being and difficulty in revealing oneself to others. On the other hand, being open-minded (e.g., open to new experiences) acted as a facilitator.

#### 3.2.3 Life circumstances

Participation barriers related to life circumstances included lack of time, private space, energy or extrinsic motives, stressful life experiences, or crises. Also, participants indicated that they had stopped using the program because of improved psychological well-being. Participants reported a lack of time for a variety of reasons, such as a heavy workload, childcare, household chores, obligations to others, social activities, travel, or difficulties with time management. Difficulties in accessing the program without a suitable and private location were also experienced. Moreover, participants often reported not using the program due to a lack of energy, e.g., feeling fatigued after a long working day. Several participants reported experiencing stressors or crises while using the program, such as illness, illness or loss of a loved one, difficult and stressful working conditions, and household stress. Finally, one participant felt a lack of extrinsic motives, such as incentives from the healthcare institution: “*Even, for example, in workplaces, to initiate some kind of hour, or not, like, five-minute periods, for example, and to organize something like that at that time.”* [P3].

In addition, some indicated that life circumstances acted as facilitators to use the program. Extrinsic motives such as information about the program from their healthcare institution, colleagues, relatives, and social media acted as facilitators to start using the program. While having free time and a suitable private place to use the program helped them to be more engaged.

#### 3.2.4 Support

Participants indicated that their experience using the program may have been influenced by the support they received from the therapist or study administrators. Some felt a lack of support from their therapist: “*Well, that's the kind of feedback that's missing... The feedback would be, well, it seems that when you're there, you could immediately ask. Or the person who is communicating with you, they would feel your well-being even the next time without asking, wouldn't they, from the behavior, from the eye contact, from the facial expressions, and all the other empirical things that, well, you just see it visually and it's... I think obviously live contact would be the best thing (laughed).”* [P3] On the other hand, some participants mentioned that they had developed a therapeutic alliance during the program, which encouraged their further involvement.

Technical support was also important for participation. One participant (P5) stated that when she had technical difficulties, the study administrator helped her to solve them which was very helpful. On the other hand, one user (P7) indicated that there was a lack of technical support while using the program. In terms of support by email or message reminders, the reminders were unsuitable for some users but suitable for others. Most participants felt that reminders to use the program were important. But few participants (P8 and P9) were dissatisfied with the reminders received: “*…in my case, these reminders make me... Aaaa works in a more stressful way (laughed)*” [P9]. For two participants, there were too many reminders (P1, P9). Meanwhile, two users indicated that there could have been more reminders (P4, P10). Lastly, one participant indicated that the timing of the reminders was unsuitable (P2).

#### 3.2.5 Content

The content of the program was also an important factor, acting either as a facilitator or as a barrier. Participants were motivated to use the program if it was perceived as professional and reliable, or if it was relevant to them and healthcare professionals in general: “*In fact, as we are now in the last few years, there are so many internal stressors, and not only internal but also external... So maybe I think it's really worth it.”* [P2]. It was also important for participants that the format of the program was appropriate. They liked the psychoeducational texts, tasks, and length of the program. In addition, it was noted as important if the program was presented in an uncomplicated and coherent way and whether the content of the program was engaging and attractive.

On the other hand, the same factors were seen differently by other participants. Usage was reduced if the content was considered unengaging, boring, or irrelevant to the user or healthcare professionals in general. Some users found the content of the program unsuitable because of an inappropriate format of communication with the therapist, tasks, redundant information, and bad timing of the program or program reminders. In addition, the content of the program was too difficult to use for some participants (unclear questions or progress, too much information). It was also noted that the program reminded them of a test situation.

#### 3.2.6 Technical aspects

Technical aspects may also have influenced the use of the program. For some participants, online access to the program was convenient and acted as a facilitator. For others, the log-in method was challenging and inconvenient, and there were some technical issues: “*Well, I was supposed to go away for two weeks, and there... Well, there's the forest, there's this... You know, there's no internet, nothing. I tried, I tried. After that, I realized that I had blocked your connection somehow. I've tried for the last time and nothing.*” [P5].

## 4 Discussion

This study specifically focused on qualitative data regarding the experiences of healthcare workers who had discontinued the use of internet-delivered stress recovery program. The aim was to get a deeper insight into the usage attrition phenomenon. The thematic analysis showed that healthcare workers who stopped using the therapist-guided program tended to have more than one reason to do so. Discontinuation was motivated by lack of time, busy pace of life, travel, fatigue, previous participation, or more program-related factors such as technical difficulties in accessing the program or simply the unsuitable nature of the program itself. The analysis of identified barriers and facilitators faced by the participants while using the program revealed the importance of both personal and program-specific factors. Personal aspects included a psychological response to the program, personal characteristics, and life circumstances. Program-related aspects included the level of support received, program content, and technical aspects. The results of the study are discussed below in more detail.

Overall, the results of our study are consistent with other qualitative studies of internet interventions. Person- or program-related facilitators or barriers to internet program use have been reported in several other studies (Arnold et al., 2020; Banerjee et al., 2017; Johansson and Andersson, 2012). However, the current study shows a small imbalance between these areas, with more program-related factors than personal aspects being identified as facilitators and more personal aspects being identified as barriers compared to program-related ones. A systematic review by Waller and Gilbody (2009) also reported that the most common reason for dropout was personal rather than because of technology or social aspects. Based on these findings, recommendations can be made at institutional and individual level, as well as for program developers and researchers, as outlined below.

Some of the most important personal factors determining engagement in online psychological support may be related to the participant's intrinsic motivation, such as the desire to help oneself or even a professional interest. The current study highlights that a lack of intrinsic motivation can act as a significant barrier for engagement in internet intervention. A previous qualitative study on treatment completers of an online program showed the importance of fostering participants' intrinsic motivation to use the program (Donkin and Glozier, 2012). Motivational interviews before ICBT program may encourage participants to use the program for more days (Soucy et al., 2021; Titov et al., 2010). On the other hand, motivational interviewing may not benefit all individuals equally (Peynenburg et al., 2022). It is therefore important to restrict motivational interviews only to those who would benefit from them, in order to avoid an additional treatment burden.

Intrinsic motivation to participate in the program can also be influenced by how the program is perceived. In the current study, some participants indicated that they found the program professional, which acted as a facilitator. However, some participants saw the program as irrelevant and unlikely to help them, which acted as a barrier. Therefore, the proper initial presentation of the program to participate may be an important aspect to consider. Similar themes emerged in a study by Banerjee et al. (2017), which found that believing that mindfulness works can motivate people to carry on. One RCT study also showed that if participants did not see the credibility of an online program based on relaxation, (i.e., that it could help them to relieve stress), it can motivate dropout (Alfonsson et al., 2016). In this context, it would be important to ensure that the internet-based psychosocial programs are presented not only in a professional manner, but also with a clear description of its mechanisms and potential benefits, which could be included in the initial call for participation and in the first module of the ICBT program, in order to foster intrinsic motivation.

In this study, we observed that a negative psychological response to the program may also lead to usage discontinuation. Some participants indicated that they experienced anxiety, self-blame, guilt, or even increased stress due to the program. Similarly, a qualitative study of an internet-based mindfulness program for healthcare workers by Banerjee et al. (2017) found emerging negative thoughts and becoming self-critical were identified as the key barriers to engaging. Greater involvement of participants in the treatment process could help reduce negative experiences. Qualitative research has shown that people who take responsibility for their treatment and attribute success to themselves benefit more (Bendelin et al., 2011). One solution to encourage participants to feel in control of their treatment is in the form of patient-driven ICBT programs. In such programs, the participant decides which intervention modules they believe they may benefit from, the pace of the program, and the amount of contact they will need with a therapist. This approach can help to increase perceived levels of control and to reduce participants' anxiety to a significantly greater extent than in a conventional ICBT program (Nissling et al., 2021). Thus, the field of internet interventions for mental health care should move towards a more patient-driven tailored approach.

User expectations are also among the personal factors that influence program use (Kazlauskas et al., 2020). Some of the participants in our study felt that the program did not meet their expectation that they would have more contact with the therapist, specifically face-to-face contact. It has been recognized that certain program users may predominantly read program content without completing the tasks/homework (Bendelin et al., 2011), thus missing out on feedback from the therapist. In the current study, it was noticed that some of the participants did not even know that feedback from a psychologist was provided during the program, even though they expressed the need for it. Therefore, it might be important for the therapist to send an introduction message to the participant before he or she has even completed the tasks of the first module. Interaction with the therapist at the very beginning of the program could encourage a positive response to therapy and lead to more positive outcomes (Haas et al., 2002; Krieger et al., 2023). In particular, guidance and the quality of guidance may become more important if the program is less suitable (Berger, 2017).

Given that research shows that ICBT programs can be beneficial, it is important to look at what works for whom. For some individuals, even a brief engagement in intervention can be sufficient to achieve the expected benefits (Bisby et al., 2023; Howard et al., 1986). It is possible to increase the likelihood of a participant receiving the right dose of treatment by as much as 12 % if the treatment is matched to face-to-face or internet-delivered, according to the patients' needs (Gonzalez Salas Duhne et al., 2022). Acceptability of internet treatment is an important factor in predicting greater engagement with the program for anxiety and depression and completing more modules (Gulliver et al., 2021). In addition, lower levels of stigma, more positive attitudes towards help-seeking and personal traits such as agreeableness have been found to lead to higher acceptability. The same is true for traditional face-to-face psychotherapy, where therapists report that their clients drop out of treatment, usually because they are not satisfied with the intervention offered or because it was not as helpful as they had hoped (Kullgard et al., 2022). Accordingly, it is important to increase the acceptability of such programs in the community of healthcare workers, which may require systemic changes, such as reducing the stigma of seeking psychological support.

In our study, we noticed that program-specific aspects can also be important in hindering or prompting the use of the program. High workload and associated fatigue were found to make it difficult to implement an internet intervention for workers in a healthcare institution. Similar results have been noted in a previous qualitative study, which showed that participants may perceive the use of the online program as difficult and demanding (Halmetoja et al., 2014). However, research suggests that the optimal dosing of a low-intensity guided treatment is 4–6 sessions (Robinson et al., 2020). It is therefore important to find a balance between the right dose of treatment to achieve effects without overburdening the user. In this context, it may also be important to take a more work-focused approach to ICBT for healthcare workers, for example by including more content on workload (e.g. Asplund et al., 2019, Asplund et al., 2023). Alternatively, given the nature of HCWs' work and time constraints, it may be important to consider ultra-brief treatment adaptations. Especially in the light of research indicating that for some individuals, <3 sessions may be enough to achieve symptom relief (Fenneli and Teasdale, 1987; Saunders et al., 2019). In addition, unsuitable timing of the program can be an important factor influencing engagement as well, as research shows that the more days that elapse between sessions, the more likely a participant is to drop out of the program (Linnet et al., 2023). Therefore, the tailored timing of the program and reminders could help reduce the burden felt by the participants and make it more easily accessible. Institutional incentives to use the program, perhaps even in the form of dedicated time during the workday, may also be important considering the findings of this study.

It is also important to find ways to make internet interventions more engaging and user-friendly. The results of this study are in line with those of Alfonsson et al. (2016), which reported that finding the ICBT treatment interesting and engaging was an important factor in helping participants complete the program. One solution to encourage engagement could be to optimize the user interface of such applications (Hentati et al., 2021). Including gamification principles in internet interventions could also potentially boost participant engagement (Cugelman, 2013). It is also important to explore other technological solutions, such as virtual reality, to find more engaging treatments (Ma et al., 2021). Given that lack of social support can act as a barrier, another way to encourage engagement would be to include a social element in such programs, allowing healthcare professionals to share their experiences with other professionals. Studies have shown the importance of social relationships in engaging with and staying in online programs, as well as determining their outcomes (Cross et al., 2022; Vöhringer et al., 2020). Participants in this qualitative study also mentioned that the inclusion of a chat forum could act as a facilitator, suggesting that it may be important to consider ways of promoting sense of community in programs for health workers.

This is to our knowledge the first qualitative study to explore reasons for usage discontinuation in an online stress recovery intervention for healthcare workers. Moreover, as the participants were informed about the qualitative study at pre-intervention assessment, it was possible to successfully recruit most of the program dropouts. Another advantage of the study is that the participants were interviewed immediately after the end of the program, which allowed us to capture their experiences less distorted by time. However, the results of this exploratory study need to be seen in the context of the limitations of qualitative research (Greenhalgh and Taylor, 1997). Firstly, the results cannot be generalized to usage attrition in internet interventions in general, as participants were healthcare workers. In addition, all participants were female, and further research is needed to evaluate the experiences of male healthcare professionals in using internet interventions. Also, the interviews were conducted by several different interviewers, therefore participants' answers may vary depending on this. Another drawback of the study is that there is a potential bias in the results, as the first author of the paper is writing a dissertation based on the study and is involved in the program efficacy trial. However, to reduce bias, an additional coder, non-program related, was included in the study. Thus, despite the limitations of the study, the results provide valuable insights into the usage attrition of internet interventions.

## 5 Conclusions

The results of this study will hopefully increase our knowledge about factors that can influence usage attrition from internet-delivered mental health interventions for healthcare workers. We recognize that there is usually no single reason for discontinuing a program, thus a holistic approach to fostering program engagement is important. The themes identified illustrate that participants face both personal and program-related facilitators and barriers to using the internet-delivered psychosocial program. The results indicate that factors that are important in face-to-face therapy may also be important in internet interventions – where unrealistic expectations and doubts about the effectiveness of treatment can lead to an early withdrawal. However, some of the methods currently used to encourage participation in such programs can also act as barriers for some people. This points to the need to move towards a more personalized and patient-driven approach to psychological treatment. Therefore, the technical features of the program should act as more engaging and stimulating depending on the needs of the client. The role of the institution behind the treatment, which ensures support and facilitates the use of internet programs for healthcare workers, is also important and should be further investigated.

## Funding

The project has received funding from 10.13039/501100008530European Regional Development Fund (project No: 01.2.2-LMT-K-718-03-0072) under grant agreement with the Research Council of Lithuania (LMTLT).

## Declaration of competing interest

The authors declare that they have no known competing financial interests or personal relationships that could have appeared to influence the work reported in this paper.

## Appendix A Supplementary data

Supplementary material 1 Image 7

Supplementary material 2 Image 8
