# Supplementary material for: Exploring reasons for usage discontinuation in an internet-delivered stress recovery intervention: A qualitative study
Source: Internet Interv. 2023 Oct 24;34:100686. doi: 10.1016/j.invent.2023.100686 (PMC10628352; doi:10.1016/j.invent.2023.100686)
Supplement: Supplementary file 1 — Supplementary material 1 [file mmc1.docx]

# SUPPLEMENTARY MATERIALS

Appendix 1. Qualitative study flow.





Appendix 2. Characteristics of the sample at baseline (*N* = 91).

| Variable | Not included  (*n* = 79) | Included  *(n* = 12) | Significance statistics |
| --- | --- | --- | --- |
| Gender |  |  |  |
| Female | 78 (98.7%) | 12 (100.0%) | χ^2^(1) = 0.15, *p* = .695 |
| Male | 1 (1.3%) | 0 (0.0%) |  |
| Age |  |  |  |
| *M* (*SD*) | 38.78 (11.19) | 44.67 (11.80) | *t*(89) = -1.68, *p* = .096 |
| Range | 19-68 | |  |
| Education |  |  |  |
| Secondary or lower | 4 (5.1%) | 1 (8.3%) | χ^2^(2) = 0.53, *p* = .767 |
| Post-secondary or vocational | 21 (26.6%) | 4 (33.3%) |  |
| Higher education | 54 (68.4%) | 7 (58.3%) |  |
| Long-term relationship |  |  |  |
| No | 20 (25.3%) | 5 (41.7%) | χ^2^(1) = 1.40, *p* = .237 |
| Yes | 59 (74.7%) | 7 (58.3%) |  |
| Position |  |  |  |
| Doctor | 23 (29.1%) | 2 (16.7%) | χ^2^(3) = 3.41, *p* = .333 |
| Resident doctor | 9 (11.4%) | 0 (0.0%) |  |
| Nurse | 23 (29.1%) | 6 (50.0%) |  |
| Other | 24 (30.4%) | 4 (33.3%) |  |
| Work status |  |  |  |
| Part-time | 6 (7.6%) | 1 (8.3%) | χ^2^(2) = 1.12, *p* = .573 |
| Full-time | 34 (43.0%) | 7 (58.3%) |  |
| > Full-time | 39 (49.4%) | 4 (33.3%) |  |
| Type of services* |  |  |  |
| Outpatient | 42 (53.2%) | 6 (50.0%) | χ^2^(1) = 0.04, *p* = .838 |
| Inpatient | 27 (34.2%) | 3 (25.0%) | χ^2^(1) = 0.40, *p* = .529 |
| Rehabilitation | 7 (8.9%) | 2 (16.7%) | χ^2^(1) = 0.71, *p* = .399 |
| Nursing | 8 (10.1%) | 5 (41.7%) | **χ^2^(1) = 8.46, *p* = .004** |
| Paramedics | 14 (17.7%0 | 0 (0.0%) | χ^2^(1) = 2.51, *p* = .113 |
| Intensive care | 6 (7.6%) | 0 (0.0%) | χ^2^(1) = 0.98, *p* = .323 |
| Work experience |  |  |  |
| < 2 years | 12 (15.2%) | 1 (8.3%) | χ^2^(3) = 1.01, *p* = .798 |
| 2-5 years | 19 (24.1%) | 2 (16.7%) |  |
| 6-10 years | 7 (8.9%) | 1 (8.3%) |  |
| > 10 years | 41 (51.9%) | 8 (66.7%) |  |
| In psychological treatment |  |  |  |
| No | 36 (45.6%) | 3 (25.0%) | χ^2^(2) = 2.17, *p* = .339 |
| Yes, earlier in life | 35 (44.3%) | 8 (66.7%) |  |
| Yes, currently attending | 8 (10.1%) | 1 (8.3%) |  |
| Taking medication due to mental health difficulties |  |  |  |
| No | 76 (96.2%) | 11 (91.7%) | χ^2^(1) = 0.51, *p* = .475 |
| Yes | 3 (3.8%) | 1 (8.3%) |  |
| Recent use of other self-help apps |  |  |  |
| No | 70 (88.6%) | 12 (100.0%) | χ^2^(1) = 1.52, *p* = .218 |
| Yes | 9 (11.4%) | 0 (0.0%) |  |
| Perceived stress *M* (*SD*) | 7.62 (2.82) | 7.00 (1.28) | *t*(30.8) = 1.28, *p* = .212 |
| Stress recovery *M* (*SD*) | 51.27 (9.33) | 52.33 (8.27) | *t*(89) = -0.37, *p* = .709 |
| Anxiety *M* (*SD*) | 2.92 (1.67) | 2.83 (0.94) | *t*(23.4) = 0.28, *p* = .785 |
| Depression *M* (*SD*) | 2.56 (1.67) | 2.75 (1.36) | *t*(89) = -0.38, *p* = .704 |
| Psychological well-being *M* (*SD*) | 41.06 (17.65) | 36.33 (15.11) | *t*(89) = 0.89, *p* = .382 |

*** Participants could choose more than one answer from the list

Appendix 3. Semi-structured interview protocol


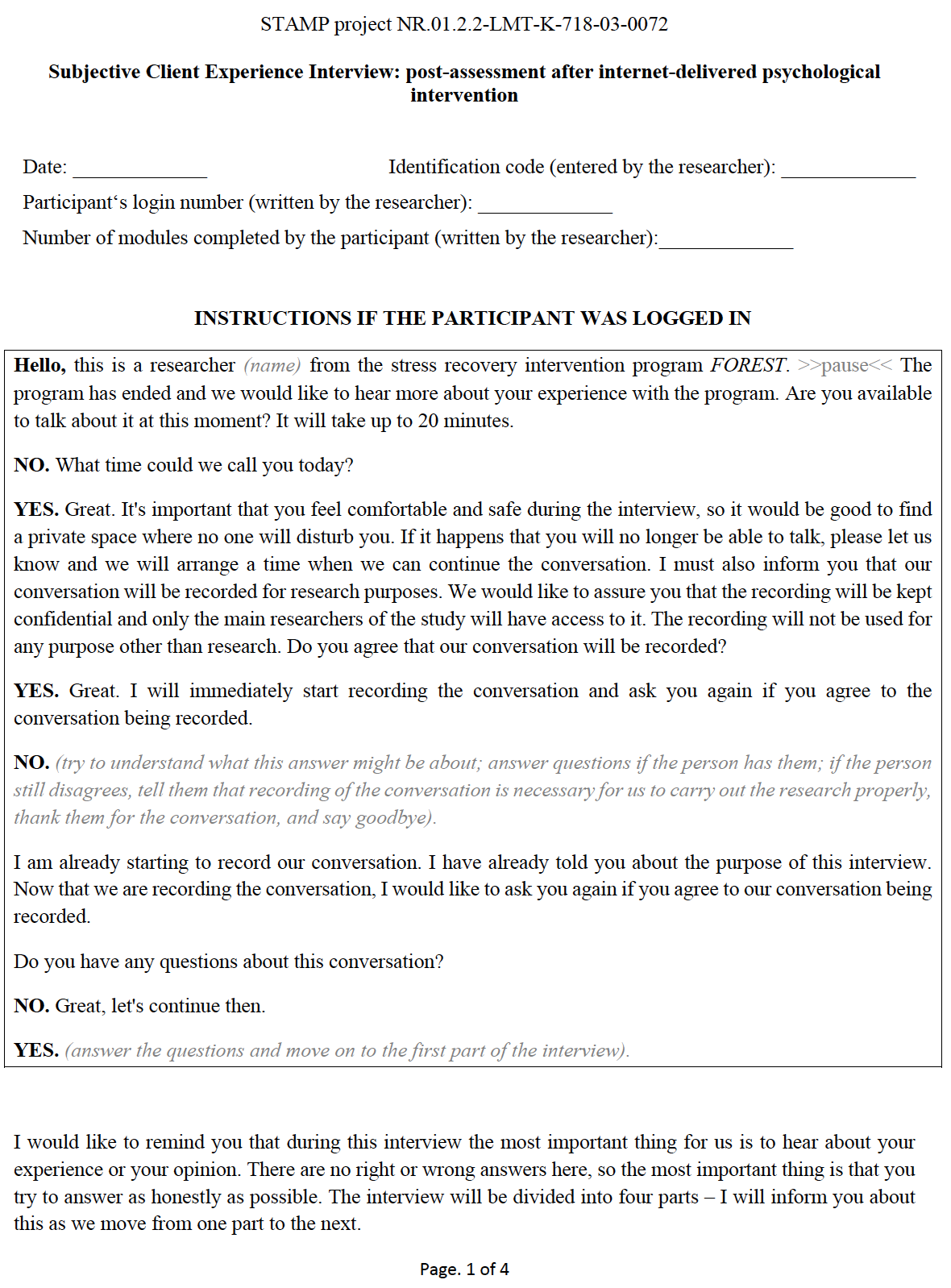


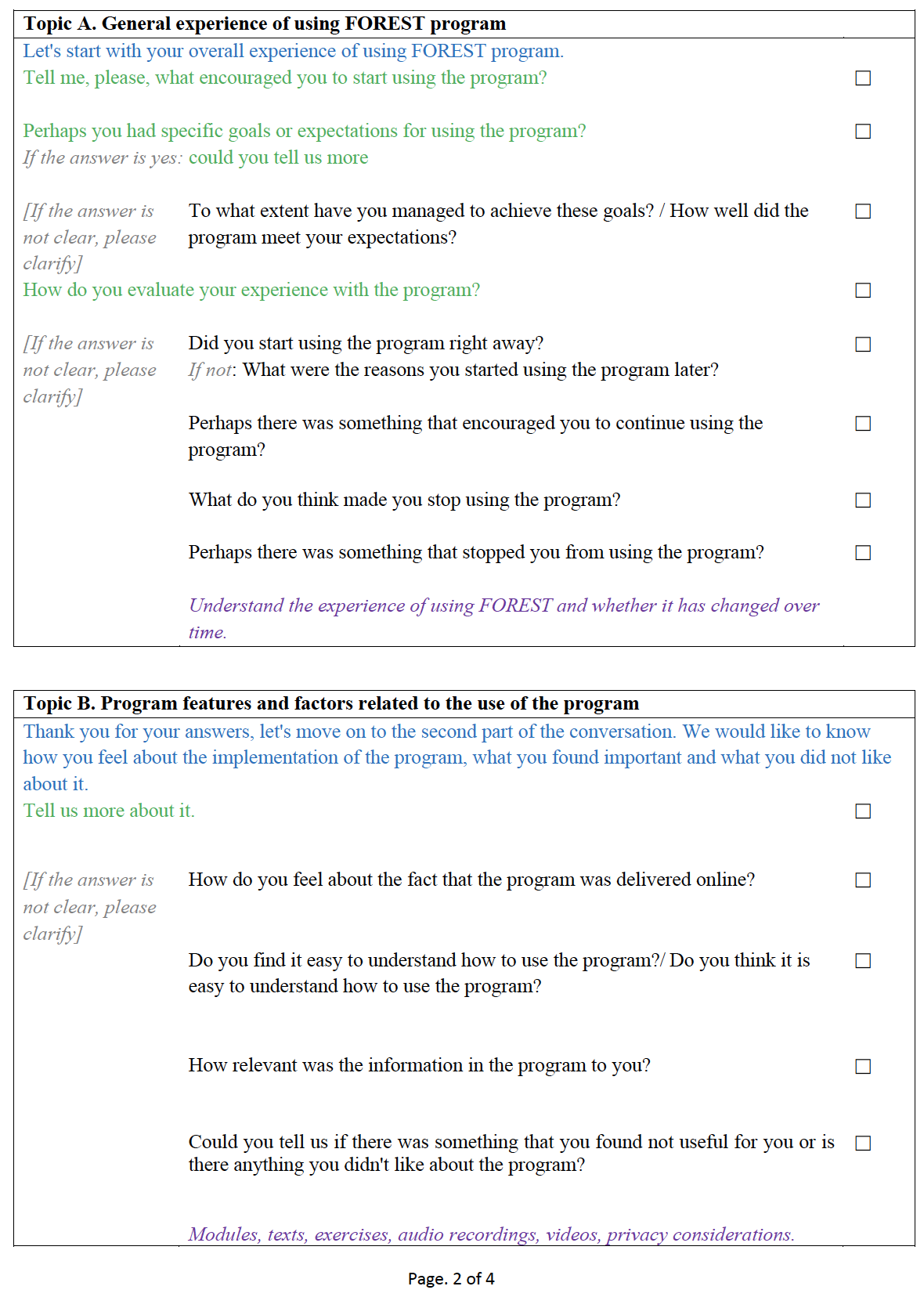


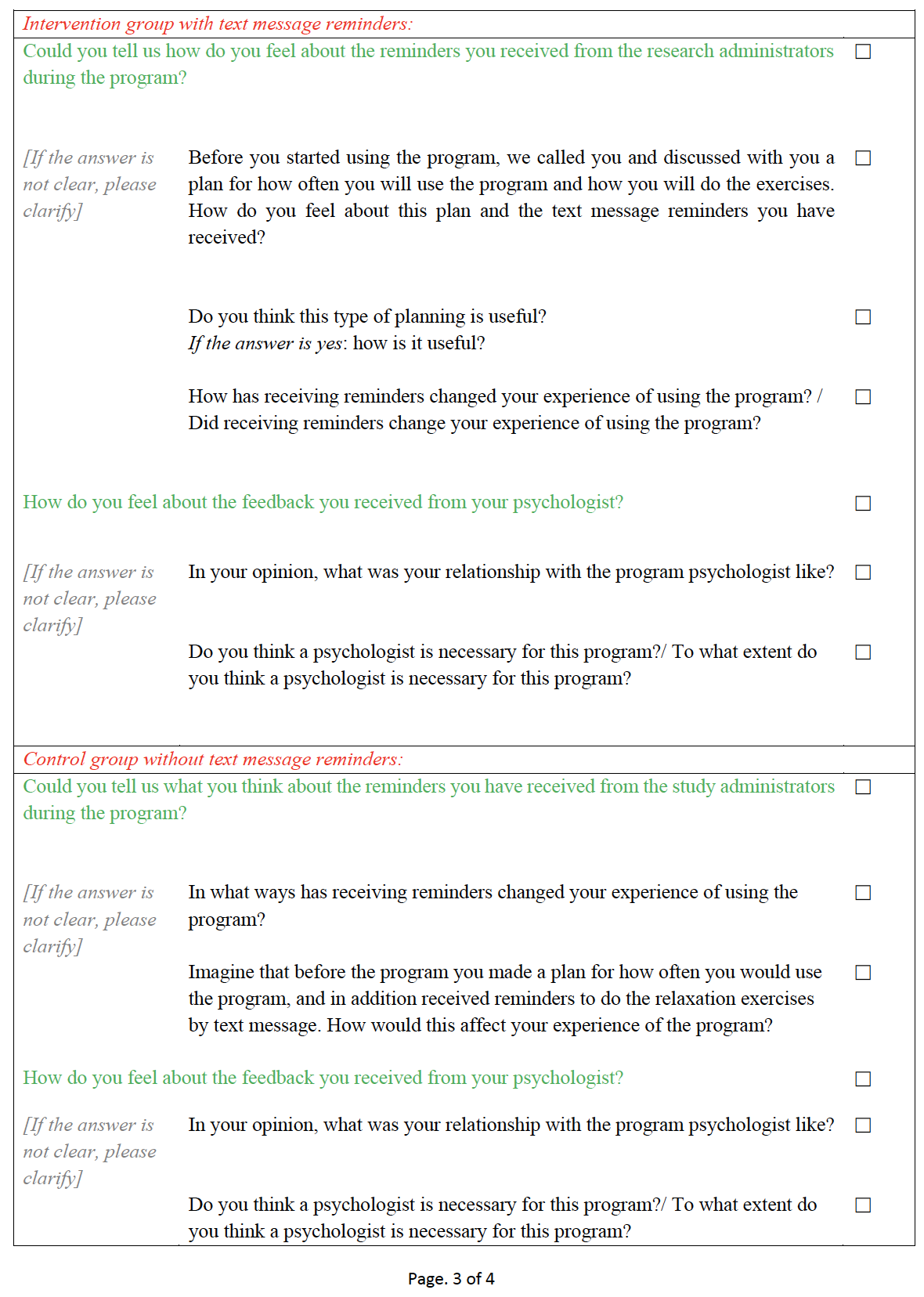


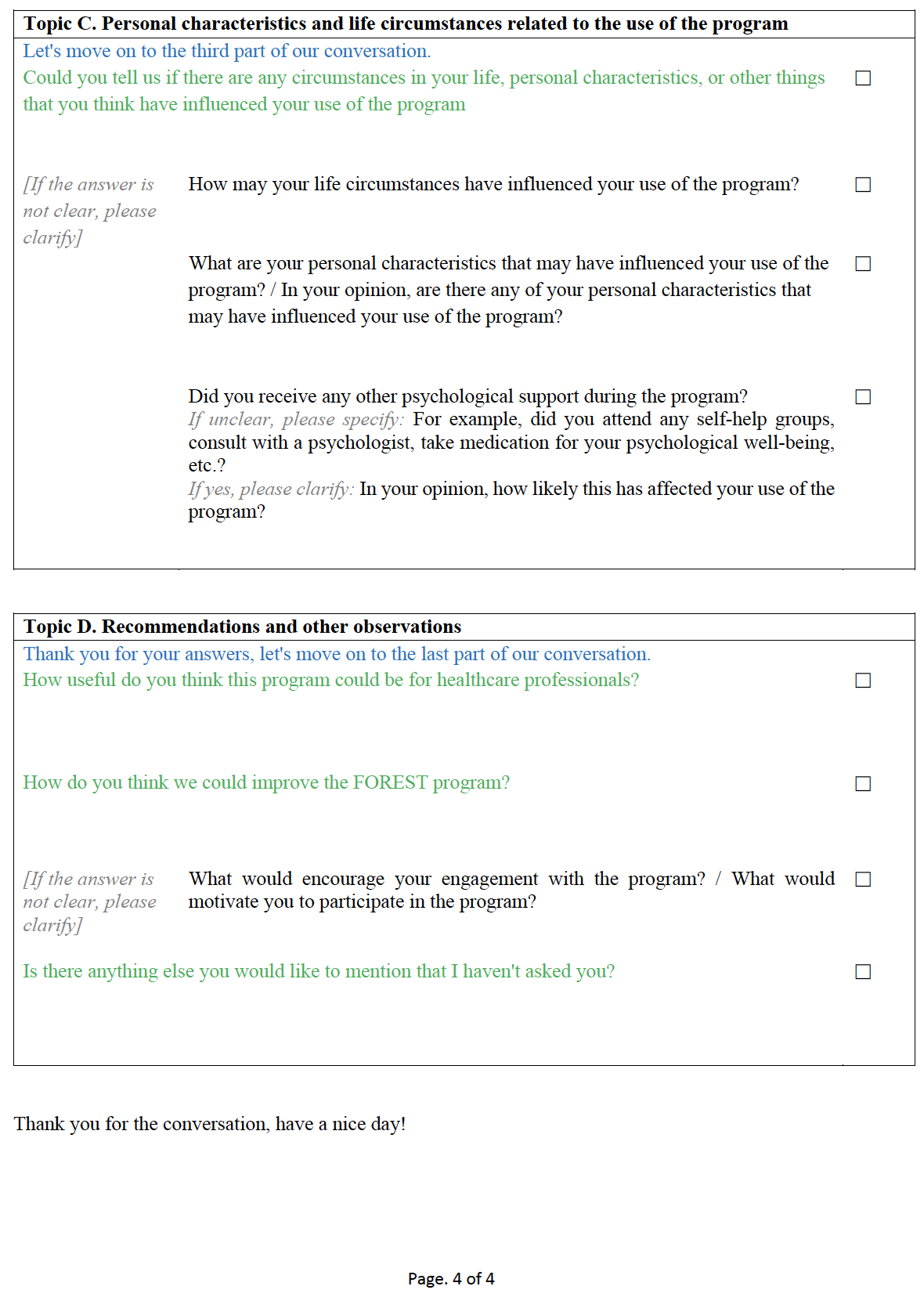


Appendix 4. Themes of qualitative interviews with subthemes and codes.

| Theme | Subtheme | Quote | Participants |
| --- | --- | --- | --- |
| Psychological response to the program | Expectations unmet (–) | *Maybe my expectations were higher and just too high. I was expecting to see someone who would explain, who would... It would be interesting to listen to, and it’s so... It's dull like a textbook.* [P6] | P2, P3, P6, P9, P10 |
|  | Negative emotions experienced (–) | *It's just the program itself, for example, it's... I associate it with my work, because it was on my workplace page and... Well just psychologically it's associated and I didn't want to just log in and I wanted to hang out with people, with my girlfriends and... And just in that other environment. To get away from everyday life completely.* [P11] | P3, P4, P5, P6, P7, P8, P9, P11 |
|  | Positive emotions experienced (+) | *And especially when there was music, like this... And I look at it and I think, my lord, how short and beautiful life is (laughed). Well, that's me... What a short and beautiful life... Even the troubles that are there, really, they are... That's what it's like even... Even when my this relative who needed help, I was so happy that I could do something good for someone.* [P5] | P2, P5, P11 |
| Personal characteristics | Self-neglect (–) | *What worries me most is that I don't take care of myself.* [P7] | P3, P4, P7, P12 |
|  | Difficulties in disclosing oneself (–) | *I am that kind of person... That's so... Sanguine like that... Well, I'm kind of sad, but I don't want to show others that I'm sad.* [P5] | P5 |
|  | Lack of intrinsic motives (–) | *Well, I just wanted to leave everything behind, to be on holiday, because I was doing a pilgrimage in Spain, Portugal, and so I didn't have the desire or the opportunity to join in there.* [P11] | P1, P2, P3, P8, P10, P11, P12 |
|  | Prior knowledge (–) | *It's just that I signed up a second time because I thought it was... That it's some sort of extension after the same thing, I was a bit lenient about it because I knew absolutely everything.* [P1] | P1, P3, P4, P7, P8 |
|  | Being open-minded (+) | *And I do a lot of things, well, in the sense that I do whatever I find, whatever is offered to me, I'm involved in everything, and I think that somehow it's good for me in some way. [P7]* | P1, P7 |
|  | Intrinsic motives (+) | *I think it started during the pandemic, and I think it's the third time that I've shared it and then I thought I should register myself and see what you're offering and what it's all about and I'd be interested in that too... In terms of information and maybe something for myself or my work.* [P8] | P3, P4, P5, P6, P8, P9, P10, P11, P12 |
| Life circumstances | Improved well-being (–) | *During the holidays I was busy every day and I relaxed very well and enjoyed it very much.* [P6] | P6, P7 |
|  | Lack of extrinsic motives (–) | *Even, for example, in workplaces, to initiate some kind of hour, or not, like, five-minute periods, for example, and to organize something like that at that time.* [P3] | P3 |
|  | Lack of time (–) | *…well, I haven't used it very much, because of the busyness, because of the busy pace, because of the lack of time, so it's... I missed a lot. So maybe, as I say, it wasn't very fulfilled, my whole thing... the whole process of the program, observation, and participation.* [P3] | P1, P2, P3, P4, P5, P6, P7, P8, P11, P12 |
|  | No private space (–) | *And even more so at home, somehow, I didn't feel like… I didn't feel like joining and going deeper. So as much as I had a spare minute at work, I would turn on and go through the program really quickly. Well, as I said, it was bothering me... I don't know, that the distance and the conditions weren't good.* [P2] | P2, P10, P11 |
|  | Lack of energy (–) | *You come home from work tired, you want to turn on the program and look at a person and listen. If you're tired, you close your eyes… but you have to sit here and read instead.* [P6] | P2, P4, P6, P7, P8, P11 |
|  | Life stressors/crises (–) | *And there, because I must visit the sick sometimes, I infected my mother, I didn't realize that I was infected myself and my mother died of corona virus. I understand, I was very aware of everything, but still to this day I still... Well, somehow that... Well, maybe (she wouldn't have?) survived*. [P7] | P1, P2, P3, P4, P5, P6, P7, P8, P9, P10, P11 |
|  | Extrinsic motives (+) | *As far as I understood from my colleague who told me everything she was also participating, but she... Well, I would have... I would have liked to have been more involved, but I was involved... As much as I could, as much as I was able to. But she also said that it's a really good program, it just needs a lot of well... Well, to realize that you have to give up everything and work here - it's good for you.* [P7] | P2, P4, P5, P6, P7, P9, P10, P12 |
|  | Having free time (+) | *Also, when I had a free day when I was already relaxed and stress-free, I would turn it on.* [P6] | P6 |
|  | Having a suitable place (+) | *…once I took some time for it there at home, in my own space, I wanted to be comfortable, and then the next time I tried to spend time in the work environment, but the environment didn't feel so relaxing…* [P10] | P1, P4, P7, P10 |
| Support | Lack of therapist support (–) | *Well, that's the kind of feedback that's missing... The feedback that would be, well,* *it seems that when you're there, you could immediately ask. Or the person who is communicating with you, they would feel your well-being even the next time without asking, wouldn't they, from the behavior, from the eye contact, from the facial expressions, and all the other empirical things that, well, you just see it visually and it's... I think obviously live contact would be the best thing (laughed).* [P3] | P1, P2, P3, P5, P10, P11 |
|  | Unsuitable number of reminders (–) | *Partly positive, because if, say, an entry comes in and I forget to look at it and I get a one-time reminder, that's fine. But when there are too many, it's a bit annoying (laughed). [P9]* | P1, P6, P8, P9, P11 |
|  | Lack of technical support (–) | *I had to make an effort to look for you, so that... Me... To receive some kind of help.* [P7] | P7 |
|  | Therapeutic alliance (+) | *Because of that personal connection, it doesn't matter that you haven't seen the person, the psychologist, but when it's written to you, you feel that kind of... Well, for you personally... You feel differently than when you read a public message.* [P4] | P1, P4, P5, P11 |
|  | Suitable number of reminders (+) | *It wasn't like "We're going to bombard you here every day" - no, no... It was just a very nice, polite way of asking how often you would like to, really…* [P3] | P1, P2, P3, P4, P5, P6, P7, P9, P10, P11, P12 |
|  | Technical support (+) | *…for example, there was... Something was there that was not clear to me, I wrote, they wrote back so and so do. Well, I did. Well, even with this login, when I had... That's it. Well, I was very pleased.* [P5] | P5 |
| Content | Unengaging content (–) | *Well, I found it too boring.* [P6] | P4, P5, P6, P11 |
|  | Irrelevant content (–) | *I think if anyone has, actually has, more serious problems, then, actually, it's very good that something like this is being done… In fact, something is being improved, that, well... I think it is really good, but... But for whoever needs it.* [P6] | P1, P3, P4, P6, P8, P9, P12 |
|  | Unsuitable format (–) | *Overall, I thought the program was a very good thing, it's just that it's not for everyone. Personally, I didn't like that universal approach at all. [P9]* | P1, P2, P3, P5, P6, P7, P8, P9, P11, P12 |
|  | Too complex (–) | *Well, maybe the first time I logged in, it was like aaa... There was a lot of text, I thought it was very, very...Text... Although maybe there wasn't much of it... I'm just talking about my impression. I mean, when I come back, tired after work, I think - "Oh, I still need to sit down there to log in to that program, because I signed up, because I'm interested"... And I log in and I see the text again and I just flicked my eyes and closed them and thought - "No. I'll look tomorrow because I'm already feeling tired'. [P8]* | P1, P3, P5, P7, P11 |
|  | Engaging content (+) | *And mindfulness that you have been posting in Youtube, I have been listening to it a lot. Sometimes I get up early in the morning and I listen, or at night I lie down and I listen until I fall asleep, that's it… [P7]* | P1, P5, P7, P8, P9 |
|  | Relevant (+) | *In fact, as we are now in the last few years, there are so many internal stressors, and not only internal but also external... So maybe I think it's really worth it. [P2]* | P1, P2, P3, P4, P5, P7, P10, P11, P12 |
|  | Suitable format (+) | *…the recording itself wasn't too long, it's a couple of minutes, so it's quite suitable and I like it. I've saved a few of the recordings and I haven't listened to them again yet, but I think I might want to one day and I will do those recordings.* [P9] | P1, P3, P4, P5, P6, P7, P9, P10, P11, P12 |
|  | Not too complex (+) | *It seems like everything is going well there, gradually going from one to the other... And also all, as I say, those corners are covered, aren't they, on some kind of anxiety or other things.* [P3] | P3, P4, P7, P9 |
|  | Professional (+) | *Amm, and as for what I liked, well, I'll repeat that the overarching is... And because it's, well, I don't know, probably... I don't know if it's a scientific work or not, but it seems to me very... If it's some kind of scientific work, it's a careful, qualitative attempt to... Well, just as it is done. And... and it's important, I imagine, that not only the project, but, in fact, it... That quality is important.* [P4] | P3, P4, P10 |
| Technical aspects | Limited access (–) | *You see, I had to go to... Well, I was supposed to go away for two weeks and there... Well, there's the forest, there's this... You know, there's no internet, nothing. I tried, I tried. After that, I realized that I had blocked your connection somehow. I've tried for the last time and nothing.* [P5] | P3, P4, P5, P7 |
|  | Technical issues (–) | *Because they sent me a message to register, I tried this way and that way and nothing worked, it wouldn’t allow me. Maybe I should have called you, but I... Work leads to work, action to action and so I didn't call and I didn't join the second part, I was in the first part.* [P7] | P1, P5, P7 |
|  | Convenient access (+) | *It’s a good thing it was online. We've learned to do a lot of things online in that time, that accessibility... (laughed) In general, it seems to be more accessible.* [P4] | P1, P2, P3, P4, P6, P7, P9, P10, P12 |

*Note*. “–“ – barriers, “+” – facilitato
